# Analysis of Vibration Characteristics for Rotating Braided Fiber-Reinforced Composite Annular Plates with Perforations

**DOI:** 10.3390/ma17225402

**Published:** 2024-11-05

**Authors:** Haibiao Zhang, Zhen Li, Shixun Wang, Tao Liu, Qingshan Wang

**Affiliations:** 1 College of Mechanical and Electrical Engineering, Central South University, Changsha 410083, China; 213702051@csu.edu.cn (H.Z.);; 2 State Key Laboratory of Precision Manufacturing for Extreme Service Performance, Central South University, Changsha 410083, China; 3 Hunan Aviation Powerplant Research Institute, Aero Engine Corporation of China, Zhuzhou 412002, China; 4 School of Civil Engineering, Central South University, Changsha 410075, China

**Keywords:** fiber-reinforced composite, coordinate mapping, annular plate with holes, forward and backward vortex frequency, differential quadrature finite element method

## Abstract

In the current study, a comprehensive numerical model for analyzing the vibrational characteristics of braided fiber-reinforced composite (BFRC) rotating annular plate with perforations under diverse boundary constraints was introduced. This model employs the differential quadrature finite element method (DQFEM), which was developed based on the first-order shear deformation theory (FSDT) and coordinate transformation approach. The BFRC material, specifically a two-dimensional biaxial orthogonal fabric, was utilized to fabricate the annular plate with two distinct types of holes: circular and sector-shaped. The model’s convergence, accuracy, numerical stability, and reliability were confirmed through comparative assessments utilizing data from the literature, from ABAQUS software, and from experimental findings. The analysis focuses on studying the influences of structural properties, material parameters, and boundary restraints on the frequencies of vibration for BFRC rotating annular plates with holes. This theoretical model helps provide scientific basis and technical guidance for the stability and lightweight design of rotating annular plates, such as rotor structures in aircraft engines.

## 1. Introduction

Annular plate structures with holes have been extensively applied in various mechanical rotating devices, including gears, compressors, turbines, and separators [1,2,3,4,5]. In order to guarantee the annular plate element possesses excellent performances of high stiffness, low weight, and good structural stability, laminated composite materials are selected to fabricate the annular plate structures. As representative examples of advanced laminated composite materials, braided fiber-reinforced composites exhibit superior properties over traditional counterparts, particularly in terms of enhanced shear stiffness, improved transverse strength, increased resistance to delamination and impacts, and enhanced damage resistance [6,7,8,9,10]. Based on the above excellent properties, the braided fiber-reinforced composite material is more suitable for structures with holes. The annular plate structures with holes fabricated by braided fiber-reinforced composite material will be applied to the mechanical rotating devices with high rotating speeds. The dynamic behaviors of the braided fiber-reinforced composite rotating annular plate structures have important influences on the global stability and reliability of mechanical rotating devices, especially for high-speed working conditions. Consequently, an investigation of the vibrational characteristics of the braided fiber-reinforced composite rotating annular plates featuring through-holes is essential to inform their design and production processes.

Numerous researchers have committed to studying the vibrational features of plates and shells with perforations. Song et al. [11] developed an exact dynamic model, applying the domain segmentation integral method (DSIM) and the virtual connecting spring procedure to forecast the vibration behaviors of plates with arbitrary shapes and diverse cut-outs under elastic restraints. Avalos et al. [12] derived the auxiliary energy function corresponding to the two perforations from the full-plate energy function, and then they solved for the first four orders of the vibration frequencies for a simply-supported rectangular plate with free-edge rectangular holes via the Rayleigh–Ritz technique. Liew et al. [13] developed an energy-based plate model with a central cut-out, utilizing L-shaped fundamental elements. They subsequently calculated the free vibrational properties of the structure by applying the Ritz technique. Chen et al. [14] employed Chebyshev polynomials with the Rayleigh–Ritz energy scheme for the numerical resolution of the in-plane and bending-free vibration in rectangular plate structures featuring diverse cut-outs. Wang et al. [15] utilized the spectral geometry method, which involves expressing the structural displacement function using an improved Fourier series expansion, to establish a numerical model for analyzing the vibrational characteristics of the rectangular plate with perforations, where the regions containing the holes were modeled with zero stiffness. Huang et al. [16] established a numerical model for a functionally graded perforated rectangular plate by employing discrete subdomains and coordinate transformations. They employed an enhanced Fourier series in conjunction with the Ritz procedure to derive the vibrational features of plates. Lee et al. [17,18,19] proposed indirect Boundary Integral Formulations (BIEMs), which involve constructing linear algebraic equations by discretizing the interpolate points on boundaries. The free vibration frequencies of a perforated circular plate were then obtained using a direct search method and singular value decomposition. Senjanović et al. [20] developed distinct potential and kinetic energy formulations for the intact and perforated sections of the circular plate. By subtracting these energies, they derived the energy functional for the perforated circular plate. Subsequently, they employed the assumed mode method to determine its vibrational properties. Maharudra et al. [21] established a finite element vibration analysis model for trapezoidal open-composite plate structures on the basis of the high-order shear deformation theory (HSDT) and the isoparametric plate element of eight-node. Sun et al. [22] constructed a vibration analysis model tailored for composite plate structures featuring various cut-outs. Do and Lee [23] derived the governing equations for holed functionally graded plates from the improved quasi-3D hybrid HSDT with four unknown variables, incorporating Hamiltonian theory. They subsequently used isogeometric analysis to solve the free vibrational characteristics of the holed plate. Noga et al. [24] adopted transverse vibration research for annular plates with various perforations by means of the finite element scheme and experimental verification. Narwariya et al. [25] analyzed the modal shapes and harmonic responses of eccentrically perforated isotropic annular plates using the finite element technique. Chen et al. [26] utilized Bessel and Fourier wave functions as trial displacement functions to investigate the vibrations of eccentric annular plates. Askari et al. [27] utilized the variable separation method and polar coordinate transformation to obtain vibrational frequencies of the eccentric annular plate.

Yang et al. [28,29] conducted research into symmetric and asymmetric buckling and vibration phenomena within rotating eccentric annular plates by means of the transformed differential quadrature procedure and conformal mapping. Lee et al. [30,31] studied the free and forced vibrations of rotating multi-layer toroidal plates, employing the layer-wise zig-zag theory within the HSDT framework. Yang et al. [32] considered geometric nonlinearities and the effects of temperature, established a free and forced vibration model for rotating graphene-platelets-reinforced composite annular plates under complex external load excitations, applying the Galerkin method along with the harmonic balance method. Qin et al. [33] used the Rayleigh–Ritz scheme to study the vibrations of cylindrical shells interfaced with plates of medium thickness. Liu et al. [34] analyzed the vibrational characteristics of discrete coupled toroidal plates-cylindrical shell structures using wave propagation methods and the virtual spring method, revealing significant modal coupling effects. Sharifi et al. [35] established a novel dynamic modeling approach for eccentric semi-annular and eccentric annular plates made of rotationally porous nanocomposites in a thermal environment. They utilized the transformed differential quadrature scheme with conformal mapping to determine the free vibrational characteristics.

Analysis of the vibrational features of plates and shells constructed from braided composite materials has garnered significant academic interest. Maji et al. [36,37,38,39] have formulated a generalized dynamic model to evaluate the vibrations of rotating 3D braiding shell and plate structures. Singh et al. [40] developed an analytical model for free vibrations and buckling analysis of the laminated and 3D woven composite plate via the triangular deformation theory and triangular-hyperbolic deformation theory, where shear strain shape functions were used to describe the boundary conditions. Huang et al. [41] explored the nonlinear dynamic features of the 3D woven composite plate, utilizing the HSDT integrated with von Kármán nonlinear kinematics, employing the enhanced perturbation approach along with the Galerkin technique. Li and Wang [42] analyzed the large-amplitude vibration of a three-dimensional braided composite cylindrical shell under axial force by means of HSDT and the two-step perturbation technique. Tsai et al. [43] conducted a simulation to investigate the impact of fabric characteristics on the elastic modulus of woven plates; the parallelogram spring model and finite element method (FEM) were employed to analyze the data.

The existing literature highlights a significant gap in the investigation of vibrational behavior in the braided fiber-reinforced composite (BFRC) rotating annular plate with holes. Consequently, this study aimed to estimate the vibration characteristics for such BFRC rotating annular plates with holes subjected to various boundary conditions. This is achieved by utilizing the differential quadrature finite element method (DQFEM), combined with the coordinate mapping technique, within the framework of the FSDT. The material chosen for the rotating annular plates with holes was a two-dimensional bidirectional orthogonal braided fiber-reinforced composite, and the analysis considered two hole configurations: circular and sector. Furthermore, the holes were evenly distributed on the surface of the plates, and boundary conditions were emulated using the penalty method. The calculated vibrational frequencies and modes of the BFRC rotating annular plate with holes were compared against experimental findings and FEM results to validate the precision, stability, and versatility of the proposed theoretical model. At last, a systematic analysis was conducted to examine the impacts of rotational speed, structural factors, and boundary restraints on the vibration features of the BFRC rotating annular plate with holes.

## 2. Problem Formulations

### 2.1. Description of the Model

**Figure 1:**
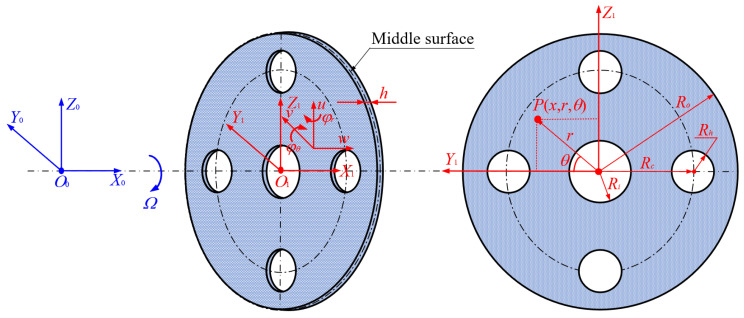
Geometrical diagram of the braided fiber-reinforced composite rotating annular plate with circular holes.

To facilitate the derivation of mathematical formulations, this study selected the braided fiber-reinforced composite (BFRC) rotating annular plate with circular holes as the subject of analysis. As one can see from Figure 1, the states of motion of BFRC rotating annular plate with circular holes were described by using two different coordinate systems named absolute coordinate system *O*_0_*X*_0_*Y*_0_*Z*_0_ (orthogonal coordinate system) and rotating coordinate system *O*_1_*X*_1_*Y*_1_*Z*_1_ (cylindrical coordinate system). The coordinate system *O*_0_*X*_0_*Y*_0_*Z*_0_ was selected to describe the position of the center of BFRC rotating annular plate with circular holes in the mechanical rotating devices, and the coordinate system *O*_1_*X*_1_*Y*_1_*Z*_1_ was chosen to describe the position of any point *P* (*x*, *r*, *θ*) located at the BFRC rotating annular plate with circular holes. The symbols *R*_o_ and *R_i_* denote the radii of the outer and inner of the BFRC rotating annular plate with circular holes, respectively. The symbols *R_c_* and *R_h_* represent the position and radius of the circular hole, and the symbol *h* indicates the thickness of the BFRC rotating annular plate with circular holes.

In accordance with the FSDT, the five displacement components—namely, *u*, *v*, *w*, *φ_r_*, and *φ_θ_*—were considered. The displacement field at any arbitrary point on the BFRC rotating annular plate with circular holes is expressed as follows.

(1) $$\begin{array}{l} \bar{u}(x,r,\theta ,t)=u(r,\theta ,t)+x\varphi_{\theta }(r,\theta ,t)\\ \bar{v}(x,r,\theta ,t)=v(r,\theta ,t)-x\varphi_{r}(r,\theta ,t)\\ \bar{w}(x,r,\theta ,t)=w(r,\theta ,t) \end{array}$$

In this equation, the variables $\bar{u}$, $\bar{v}$, and $\bar{w}$ correspond to the displacements in the *Z*, *Y*, and *X* orientations of arbitrary coordinates on the BFRC rotating annular plate with holes, whereas *u*, *v*, and *w* represent the translational displacement along the *Z*, *Y*, and *X* axes of the middle surface projection point associated with any point on the BFRC rotating annular plate with holes, and *φ_r_* and *φ_θ_* signify the rotations about the *Z* and *Y* axes of the aforementioned projection point. The strains of the arbitrary point on the BFRC rotating annular plate can be expressed as below.

(2) $$\begin{array}{l} \varepsilon_{r}=\varepsilon_{r}^{0}+x\chi_{r}^{0};\varepsilon_{\theta }=\varepsilon_{\theta }^{0}+x\chi_{\theta }^{0}\\ \gamma_{r\theta }=\gamma_{r\theta }^{0}+x\chi_{r\theta }^{0};\gamma_{rx}=\frac{\partial w}{\partial r}+\varphi_{\theta };\gamma_{\theta x}=\frac{1}{r}\frac{\partial w}{\partial \theta }-\varphi_{r} \end{array},$$

where the symbols $\varepsilon_{r}$, $\varepsilon_{\theta }$, $\gamma_{r\theta }$, $\gamma_{rx}$, and $\gamma_{\theta x}$ strain components of arbitrary point of BFRC rotating annular plate with holes. The symbols $\varepsilon_{r}^{0}$, $\varepsilon_{\theta }^{0}$, $\gamma_{r\theta }^{0}$, $\chi_{r}^{0}$, $\chi_{\theta }^{0}$, and $\chi_{r\theta }^{0}$ represent the membrane strain components. Their respective expressions are provided below.

(3) $$\begin{array}{l} \varepsilon_{r}^{0}=\frac{\partial u}{\partial r};\chi_{r}^{0}=\frac{\partial \varphi_{\theta }}{\partial r}\\ \varepsilon_{\theta }^{0}=\frac{1}{r}\frac{\partial v}{\partial \theta }+\frac{u}{r};\chi_{\theta }^{0}=-\frac{1}{r}\frac{\partial \varphi_{r}}{\partial \theta }+\frac{\varphi_{\theta }}{r}\\ \gamma_{r\theta }^{0}=\frac{1}{r}\frac{\partial u}{\partial \theta }+\frac{\partial v}{\partial r}-\frac{v}{r};\chi_{r\theta }^{0}=\frac{1}{r}\frac{\partial \varphi_{\theta }}{\partial \theta }-\frac{\partial \varphi_{r}}{\partial r}+\frac{\varphi_{r}}{r} \end{array}$$

The constitutive equation between stresses and strains of arbitrary points of BFRC rotating annular plates with holes can be determined using the following form on the basis of generalized Hook’s law.

(4) $$\left[\begin{array}{c} \sigma_{r}\\ \sigma_{\theta }\\ \sigma_{r\theta }\\ \tau_{rx}\\ \tau_{\theta x} \end{array}\right]=\left[\begin{array}{ccccc} Q_{11} & Q_{12} & 0 & 0 & 0\\ Q_{21} & Q_{22} & 0 & 0 & 0\\ 0 & 0 & Q_{66} & 0 & 0\\ 0 & 0 & 0 & Q_{44} & 0\\ 0 & 0 & 0 & 0 & Q_{55} \end{array}\right]\left[\begin{array}{c} \varepsilon_{r}\\ \varepsilon_{\theta }\\ \gamma_{r\theta }\\ \gamma_{rx}\\ \gamma_{\theta x} \end{array}\right],$$

in which the symbols $\sigma_{r}$, $\sigma_{\theta }$, and $\sigma_{r\theta }$ depict the normal stress component, respectively, and $\tau_{rx}$ and $\tau_{\theta x}$ indicate the shear stress components, respectively. The symbols *Q_ij_*(1,2,4,5,6) represent the elastic coefficients, which depend on the material property parameters. For isotropic material, the specific expressions of elastic coefficients *Q_ij_* can be written as follows:

(5) $$\begin{array}{l} Q_{11}=Q_{22}=\frac{E}{1-\mu^{2}};Q_{12}=Q_{21}=\frac{\mu E}{1-\mu^{2}}\\ Q_{44}=Q_{55}=Q_{66}=\frac{E}{2\left(1-\mu^{2}\right)} \end{array},$$

where the symbols *E* and *μ* represent Young’s modulus and Poisson’s ratio separately. For two-dimensional bidirectional orthogonal braided fiber-reinforced composite material, the elastic coefficient expression *Q_ij_* can be ascertained as follows, according to the existing literature [44].

(6) $$\begin{array}{l} Q_{11}=\frac{E_{11}}{1-\mu_{12}\mu_{21}};Q_{22}=\frac{E_{22}}{1-\mu_{12}\mu_{21}};Q_{12}=\frac{\mu_{21}E_{11}}{1-\mu_{12}\mu_{21}};Q_{21}=\frac{\mu_{12}E_{22}}{1-\mu_{12}\mu_{21}}\\ Q_{44}=G_{23};Q_{55}=G_{13};Q_{66}=G_{12};E_{11}\mu_{21}=E_{22}\mu_{12} \end{array},$$

where the symbols *E_ij_*, *μ_ij_*, and *G_ij_* separately express the coefficients with respect to flexural modulus, Poisson’s ratio, and shear modulus of BFRC materials. As reported in the existing literature [44], the specific expressions for *E_ij_*, *μ_ij_*, and *G_ij_* were determined through two-step homogenization. This study linearly fits the curve in Figure 7 of the literature [44] to determine the material parameters, as presented below:

(7) $$\begin{array}{l} E_{11}=E_{22}=105V_{f}+3.6\\ G_{12}=3.5V_{f}+1.69;G_{13}=G_{23}=3.5V_{f}+0.98\\ \mu_{12}=\mu_{21}=-0.05V_{f}+0.1639\\ \rho =560V_{f}+1200 \end{array},$$

where the symbol *V_f_* denotes the volume fraction for the total fiber of BFRC materials. The unit of elastic and shear modulus are GPa, and the unit of density is kg/m^3^. The formulation for the potential energy of a BFRC rotating annular plate with holes is derived and presented below:

(8) $$U=\frac{1}{2}\int_{-\frac{h}{2}}^{\frac{h}{2}}\int_{0}^{2\pi }\int_{R_{0}}^{R_{1}}\left(\sigma_{r}\varepsilon_{r}+\sigma_{\theta }\varepsilon_{\theta }+\sigma_{r\theta }\gamma_{r\theta }+\kappa \tau_{rx}\gamma_{rx}+\kappa \tau_{\theta x}\gamma_{\theta x}\right)rdrd\theta dx,$$

where the symbol *κ* = 5/6 is the shear correction coefficient. In accordance with the principles governing multi-body dynamics, the position vector of arbitrary points of BFRC rotating annular plates with holes is determined as follows:

(9) $$r_{P}=R_{10}+U_{P}A_{\Omega },$$

where the symbol ***R***_10_ represents the position vector of origin points of the rotating coordinate system in the absolute coordinate system; ***U****_P_* indicates the position vector of any point on the BFRC rotating annular plate with holes within the rotating coordinate system; and symbol ***A****_Ω_* denotes the coordinate transform matrix between the absolute and rotating coordinate systems. The specific expressions for ***R****_O_*_1_, ***U****_P_*, and ***A****_Ω_* are detailed below:

(10) $$\begin{array}{l} U_{P}={\left[\begin{array}{c} x\\ r\cos\theta \\ r\sin\theta \end{array}\right]}^{T}+{\left[\begin{array}{c} w\\ \left(u+x\varphi_{\theta }\right)\cos\theta -\left(v-x\varphi_{r}\right)\sin\theta \\ \left(u+x\varphi_{\theta }\right)\sin\theta +\left(v-x\varphi_{r}\right)\cos\theta \end{array}\right]}^{T}\\ A_{\Omega }=\left[\begin{array}{ccc} 1 & 0 & 0\\ 0 & \cos\Omega t & \sin\Omega t\\ 0 & -\sin\Omega t & \cos\Omega t \end{array}\right] \end{array},$$

where the symbol *Ω* denotes the rotating speed. The kinetic energy function for BFRC rotating annular plates with holes can be presented below:

(11) $$\begin{array}{l} T=\frac{1}{2}\rho \int_{-\frac{h}{2}}^{\frac{h}{2}}\int_{0}^{2\pi }\int_{R_{0}}^{R_{1}}{\dot{r}}_{P}{\dot{r}}_{P}^{T}rdrd\theta dx\\ {\dot{r}}_{P}=\left({\dot{R}}_{O1}+{\dot{U}}_{P}\right)A_{\Omega }+\left(R_{O1}+U_{P}\right){\dot{A}}_{\Omega } \end{array},$$

where the symbol denotes the density of the BFRC rotating annular plate with holes.

### 2.2. Domain Decomposition and Coordinate Mapping

**Figure 2:**
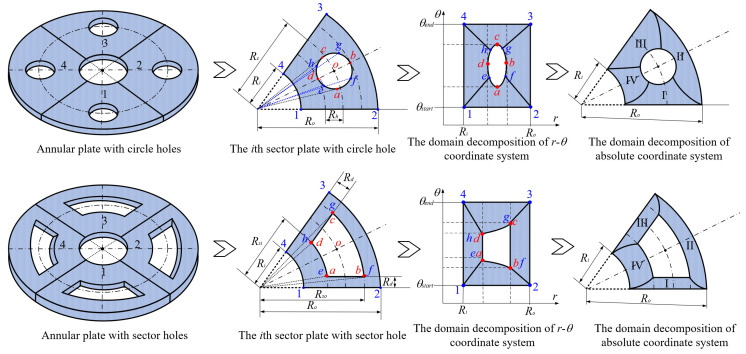
Domain decomposition of the braided fiber-reinforced composite rotating annular plate with holes.

As mentioned above, two kinds of hole structures, including circle holes and sector holes, were taken into account. The BFRC rotating annular plate needs to be decomposed into *N_p_* sector plate elements with holes uniformly according to the number of *N* holes of the BFRC rotating annular plate. Every sector plate element with a hole needs to be decomposed into four domains according to the geometrical shape of the hole. Take *N_p_* = 4 as an example; the process of domain decomposition of the BFRC rotating annular plate with perforations is shown in Figure 2.

For the BFRC rotating annular plate with circle holes, the *i*-th sector plate with a circle hole is selected as an object. *R_i_* and *R_o_* represent the internal and external radii of the *i*-th sector plate element; *θ_start_* and *θ_end_* represent the start and end angles of the *i*-th sector plate. The symbols *a*, *b*, *c*, and *d* indicate extreme points along *r* and *θ* directions in the *r*-*θ* coordinate system; the coordinates of extreme points can be expressed as follows.

(12) $$\begin{array}{l} \left(r_{a},\theta_{a}\right)=\left(\sqrt{R_{c}^{2}-R_{h}^{2}},\frac{\theta_{end}-\theta_{start}}{2}-a\sin\frac{R_{h}}{\sqrt{R_{c}^{2}-R_{h}^{2}}}\right)\\ \left(r_{b},\theta_{b}\right)=\left(R_{c}+R_{h},\frac{\theta_{end}-\theta_{start}}{2}\right)\\ \left(r_{c},\theta_{c}\right)=\left(\sqrt{R_{c}^{2}-R_{h}^{2}},\frac{\theta_{end}-\theta_{start}}{2}+a\sin\frac{R_{h}}{\sqrt{R_{c}^{2}-R_{h}^{2}}}\right)\\ \left(r_{d},\theta_{d}\right)=\left(R_{c}-R_{h},\frac{\theta_{end}-\theta_{start}}{2}\right) \end{array}$$

In order to guarantee the precision of subsequent coordinate mapping, the selection of domain decomposition points *e*, *f*, *g*, and *h* should be as far from the location of the extreme point as much as possible. However, the decomposition points can be determined according to the extreme points, and the coordinate *r* of domain decomposition points can be written as follows.

(13) $$r_{e}=r_{g}=\frac{\sqrt{R_{c}^{2}-R_{h}^{2}}+R_{c}-R_{h}}{2};r_{f}=r_{h}=\frac{\sqrt{R_{c}^{2}-R_{h}^{2}}+R_{c}+R_{h}}{2}$$

The coordinate *θ* of domain decomposition points can be determined by the coordinate *r* according to cosine law. Four domains of the *i*-th sector plate with a circle hole can be determined according to the domain decomposition points *e*, *f*, *g*, and *h* in the *r*-*θ* coordinate system. For curves *ef* and *gh*, the expressions can be written as follows due to the extreme points *a* and *c* caused by the variation of *θ*.

(14) $$\theta_{ef}/\theta_{gh}=\frac{\theta_{\mathrm{end}}-\theta_{\mathrm{start}}}{2}\mp a\cos\frac{R_{c}^{2}+r^{2}-R_{h}^{2}}{2R_{c}r}$$

For curves *fg* and *eh*, the expressions can be written as follows due to the extreme points *b* and *d* caused by the variation of *r*.

(15) $$r_{fg}/r_{eh}=R_{c}\cos\left(\left|\frac{\theta_{\mathrm{end}}-\theta_{\mathrm{start}}}{2}-\theta \right|\right)\pm \sqrt{R_{h}^{2}-R_{c}^{2}\sin^{2}\left(\left|\frac{\theta_{\mathrm{end}}-\theta_{\mathrm{start}}}{2}-\theta \right|\right)}$$

For the BFRC rotating annular plate with sector holes, the *i*-th sector plate with sector hole is chosen as object. The symbols *R_si_* and *R_so_* denote the inner and outer radii for the sector hole; the symbol *R_d_* represents the distance between the sides of the sector hole and the sector plate. The symbols *a*, *b*, *c*, and *d* indicate corner points along *r* and *θ* directions in the *r*-*θ* coordinate system; the coordinates of corner points can be written as follows.

(16) $$\begin{array}{l} \left(r_{a},\theta_{a}\right)=\left(R_{si},a\sin\frac{R_{d}}{R_{si}}\right)\\ \left(r_{b},\theta_{b}\right)=\left(R_{so},a\sin\frac{R_{d}}{R_{so}}\right)\\ \left(r_{c},\theta_{c}\right)=\left(R_{so},\frac{\theta_{end}-\theta_{start}}{2}-a\sin\frac{R_{d}}{R_{so}}\right)\\ \left(r_{d},\theta_{d}\right)=\left(R_{si},\frac{\theta_{end}-\theta_{start}}{2}-a\sin\frac{R_{d}}{R_{si}}\right) \end{array}$$

Due to the sector hole having no existing extreme points in the *r*-*θ* coordinate system, the selection of domain decomposition points *e*, *f*, *g*, and *h* is not constrained. For convenience, the domain decomposition points *e*, *f*, *g*, and *h* are assumed to be consistent with four boundary corner points: *a*, *b*, *c*, and *d*. Four domains of the *i*-th sector plate with sector hole can be ascertained according to the domain decomposition points *e*, *f*, *g*, and *h* in the *r*-*θ* coordinate system. For curves *ef* and *gh*, the expressions can be written as follows.

(17) $$\theta_{ef}=a\sin\frac{R_{d}}{r};\theta_{gh}=\frac{\theta_{\mathrm{end}}-\theta_{\mathrm{start}}}{2}-a\sin\frac{R_{d}}{r}$$

For curves *fg* and *eh*, the expressions can be written as follows:

(18) $$r_{fg}=R_{so};r_{eh}=R_{si}$$

**Figure 3:**
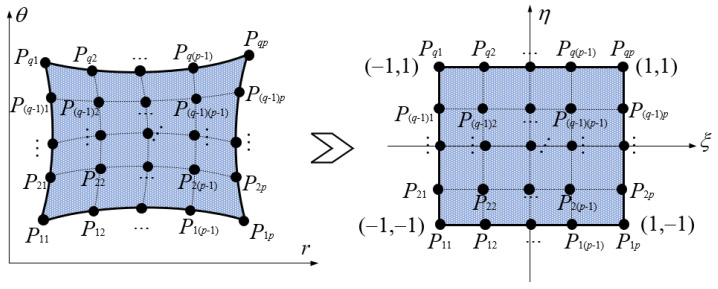
Coordinate mapping.

According to the above domain decomposition, the *i*th sector plate with a hole is decomposed into four domains, and every domain is deemed an irregular quadrilateral plate structure. To simplify the derivation of the energy expression for irregular quadrilateral plate structures, it is necessary to convert the structure into a regular square plate via coordinate mapping. The process of coordinate transformation is described in Figure 3.

As described in Figure 3, the structure of the irregular quadrilateral plate contains *p* × *q* coordinate mapping points, and the coordinate mapping points only reveal the precision of coordinate mapping. The symbol *P_ij_* denotes the mapping point. The above coordinate mapping can be regarded as the coordinate transformation between the *r*-*θ* coordinate system and *ξ*-*η* coordinate system; the corresponding coordinate transformation relationship can be written as follows:

(19) $$\begin{array}{l} r=\psi_{qp}R_{qp};\theta =\psi_{qp}\Theta_{qp}\\ \psi_{qp}=\left[\varphi_{11},\varphi_{12},\cdots ,\varphi_{1p},\cdots ,\varphi_{i1},\varphi_{i2},\cdots ,\varphi_{ip},\cdots ,\varphi_{q1},\varphi_{q2},\cdots ,\varphi_{qp}\right]\\ R_{qp}={\left[r_{11},r_{12},\cdots ,r_{1p},\cdots ,r_{i1},r_{i2},\cdots ,r_{ip},\cdots ,r_{q1},r_{q2},\cdots ,r_{qp}\right]}^{T}\\ \Theta_{qp}={\left[\theta_{11},\theta_{12},\cdots ,\theta_{1p},\cdots ,\theta_{i1},\theta_{i2},\cdots ,\theta_{ip},\cdots ,\theta_{q1},\theta_{q2},\cdots ,\theta_{qp}\right]}^{T} \end{array},$$

in which the symbols *r_ij_* and *θ_ij_* denote the coordinate component of mapping points in the *r*-*θ* coordinate system, respectively. The symbol *φ_ij_* expresses the mapping function with respect to the mapping point; the concrete expression is presented below as reported in the existing literature [45].

(20) $$\varphi_{ij}\left(\xi ,\eta \right)=\left(\prod_{s=1,s\neq i}^{p}\frac{\xi -\xi_{s}}{\xi_{ij}-\xi_{s}}\right)\left(\prod_{l=1,l\neq j}^{q}\frac{\eta -\eta_{l}}{\eta_{ij}-\eta_{l}}\right)-1\leq \xi ,\eta \leq 1$$

Based on the above coordinate mapping, the partial derivatives of the displacement function *f* regarding *r* and *θ* can be derived as follows.

(21) $$\begin{array}{l} \frac{\partial f}{\partial r}=\frac{1}{J}\left(\frac{\partial \theta }{\partial \eta }\frac{\partial f}{\partial \xi }-\frac{\partial \theta }{\partial \xi }\frac{\partial f}{\partial \eta }\right),\frac{\partial f}{\partial \theta }=\frac{1}{J}\left(-\frac{\partial r}{\partial \eta }\frac{\partial f}{\partial \xi }+\frac{\partial r}{\partial \xi }\frac{\partial f}{\partial \eta }\right)\\ J=\frac{\partial r}{\partial \xi }\frac{\partial \theta }{\partial \eta }-\frac{\partial \theta }{\partial \xi }\frac{\partial r}{\partial \eta } \end{array}$$

The actual expressions for $\frac{\partial r}{\partial \xi }$, $\frac{\partial r}{\partial \eta }$, $\frac{\partial \theta }{\partial \xi }$, and $\frac{\partial \theta }{\partial \eta }$ can be expressed as follows.

(22) $$\frac{\partial r}{\partial \xi }=\frac{\partial \sum_{ij=11}^{pq}r_{ij}\varphi_{ij}}{\partial \xi };\frac{\partial r}{\partial \eta }=\frac{\partial \sum_{ij=11}^{pq}r_{ij}\varphi_{ij}}{\partial \eta };\frac{\partial \theta }{\partial \xi }=\frac{\partial \sum_{ij=11}^{pq}\theta_{ij}\varphi_{ij}}{\partial \xi };\frac{\partial \theta }{\partial \eta }=\frac{\partial \sum_{ij=11}^{pq}\theta_{ij}\varphi_{ij}}{\partial \eta }$$

### 2.3. Differential Quadrature Finite Element Method

In accordance with the differential quadrature finite element method (DQFEM), within the natural coordinate system *ξ*-*η*, the function of displacement *f*(*ξ*, *η*) for a regular square plate element is formulated using two-dimensional weighted coefficient matrices for differential and integral. The partial derivatives for the displacement function *f*(*ξ*, *η*) relating to *ξ* and *η* are presented in the subsequent format:

(23) $$\begin{array}{l} \frac{\partial f\left(\xi ,\eta \right)}{\partial \xi }=A_{02}f;\frac{\partial f\left(\xi ,\eta \right)}{\partial \eta }=B_{02}f;\frac{\partial^{2}f\left(\xi ,\eta \right)}{\partial \xi \partial \eta }=A_{02}B_{02}f;\int_{-1}^{1}\int_{-1}^{1}f\left(\xi ,\eta \right)d\xi d\eta =C_{02}f\\ f={\left[\begin{array}{cccccc} {\tilde{f}}_{1} & {\tilde{f}}_{2} & \cdots & {\tilde{f}}_{m} & \cdots & {\tilde{f}}_{M} \end{array}\right]}^{T};{\tilde{f}}_{m}=\left[\begin{array}{cccccc} f_{m1} & f_{m2} & \cdots & f_{mn} & \cdots & f_{mN} \end{array}\right] \end{array},$$

in which ***A***_02_ and ***B***_02_ represent the differentially weighted coefficient matrices with respect to *ξ* and *η*; **C**_02_ indicates the integral weighting coefficient matrix, and the specific expressions with respect to ***A***_02_, ***B***_02_, and ***C***_02_ can be seen in the literature [46]. *M* and *N* denote the numbers of divided differential nodes in the *ξ* and *η* direction. However, for the irregular quadrilateral plate element in the *r*-*θ* coordinate system, The partial derivatives of the displacement function *f*(*r*,*θ*) relating to *r* and *θ* are given as the subsequent format according to Equation (20).

(24) $$\begin{array}{l} \frac{\partial f\left(r,\theta \right)}{\partial r}=\frac{1}{J}\circ \left(\theta_{\eta }\circ A_{02}-\theta_{\xi }\circ B_{02}\right)f=A_{2}f;\frac{\partial f\left(r,\theta \right)}{\partial \theta }=\frac{1}{J}\circ \left(-r_{\eta }\circ A_{02}+r_{\xi }\circ B_{02}\right)f=B_{2}f\\ \int_{-1}^{1}\int_{-1}^{1}f\left(r,\theta \right)drd\theta =J\circ C_{02}=C_{2}f\\ \alpha ={\left[\begin{array}{cccccc} {\tilde{\alpha }}_{1} & {\tilde{\alpha }}_{2} & \cdots & {\tilde{\alpha }}_{m} & \cdots & {\tilde{\alpha }}_{M} \end{array}\right]}^{T};{\tilde{\alpha }}_{m}=\left[\begin{array}{cccccc} \frac{\partial \alpha_{m1}}{\partial \beta } & \frac{\partial \alpha_{m2}}{\partial \beta } & \cdots & \frac{\partial \alpha_{mn}}{\partial \beta } & \cdots & \frac{\partial \alpha_{mN}}{\partial \beta } \end{array}\right]\\ J={\left[\begin{array}{cccccc} {\tilde{J}}_{1} & {\tilde{J}}_{2} & \cdots & {\tilde{J}}_{m} & \cdots & {\tilde{J}}_{M} \end{array}\right]}^{T};{\tilde{J}}_{m}=\left[\begin{array}{cccccc} J_{m1} & J_{m2} & \cdots & J_{mn} & \cdots & J_{mN} \end{array}\right] \end{array},$$

where the symbol ∘ indicates the Hadamard products; the symbols *A*_2_ and *B*_2_ denote the revised weighting coefficient matrices with regard to *r* and *θ*; and the symbol C_2_ denotes the revised integral weighting coefficient matrix. As mentioned above, the BFRC rotating annular plate with holes is decomposed as *N_p_* sector plate elements, and every sector plate element is decomposed as four domains composed of an irregular quadrilateral plate structure. The energy expression of BFRC rotating annular plates with holes is decomposed as follows.

(25) $$T=\sum_{e=1}^{N_{p}}\sum_{s=1}^{4}T_{e}^{s};U=\sum_{e=1}^{N_{p}}\sum_{s=1}^{4}U_{e}^{s}$$

Based on the DQFEM, the formulations of kinetic energy and potential energy with respect to irregular quadrilateral plate structures are written in the following matrix form:

(26) $$\begin{array}{l} T_{e}^{s}=\frac{1}{2}{\dot{u}}_{e}^{sT}M_{e}^{s}{\dot{u}}_{e}^{s}+\frac{1}{2}{\dot{u}}_{e}^{sT}G_{e}^{s}u_{e}^{s}+\frac{1}{2}u_{e}^{sT}C_{e}^{s}u_{e}^{s};U_{e}^{s}=\frac{1}{2}{\dot{u}}_{e}^{sT}K_{e}^{s}{\dot{u}}_{e}^{s}\\ e=1,2,\cdots ,N_{p};s=1,2,3,4 \end{array},$$

where the symbols $M_{e}^{s}$, $G_{e}^{s}$, $C_{e}^{s}$, and $K_{e}^{s}$ denote the mass matrix, gyroscopic matrix, damping matrix, and stiffness matrix with regard to irregular quadrilateral plate structure, respectively. The symbol $u_{e}^{s}$ indicates the displacement column vector. The concrete expressions of $M_{e}^{s}$, $G_{e}^{s}$, $C_{e}^{s}$, and $K_{e}^{s}$ can be seen in Appendix A, Appendix B, Appendix C and Appendix D. The global dynamic equations for BFRC rotating annular plates with holes can be determined by incorporating the previously established energy expressions into the Lagrange framework:

(27) $$M\ddot{u}+G\dot{u}+\left(K-C\right)u=0,$$

where the symbols ***M***, ***G***, ***K***, and ***C*** denote the global mass matrix, gyroscopic matrix, stiffness matrix, and damping matrix with respect to the BFRC rotating annular plate with holes. Boundary conditions for BFRC rotating annular plates with holes are incorporated using the penalty function method, which selectively influences the stiffness matrix ***K*** of the BFRC rotating annular plate with holes:

(28) $$\bar{K}=K+SC^{T}C,$$

where *S* represents the penalty factor and ***C*** indicates the coefficient matrix, which depends on the boundary condition, and $\bar{K}$ represents the revised stiffness matrix. Equation (27) facilitates the computation of the vibration characteristics for BFRC rotating annular plates with holes under diverse boundary conditions.

## 3. Numerical Example and Discussion

According to the above introduction, it is not hard to find that the theoretical model with respect to the vibration characterization of BFRC rotating annular plates with holes has been established through a series of formulation derivations. Initially, this present study conducted a convergence study of the developed model to ascertain the finest number of differential nodes and the value of the penalty factor, as discussed subsequently. Subsequently, model validation was performed to ensure accuracy, numerical stability, and reliability by comparing results from experiments and the literature. Ultimately, the vibrational features of BFRC rotating annular plates with holes were analyzed, examining how material parameters, structural parameters, and boundary conditions influence their natural frequency. Unless otherwise specified, the BFRC material parameters are uniformly assumed to be *E*_11_= *E*_22_ = 33 GPa, *μ*_12_ = *μ*_21_ = 0.1499, *G*_12_ = 2.67 GPa, *G*_13_ = *G*_23_ = 1.96 GPa, and *ρ* = 1356.8 kg/m^3^ in the following discussion.

### 3.1. Convergence Analysis

**Figure 4:**
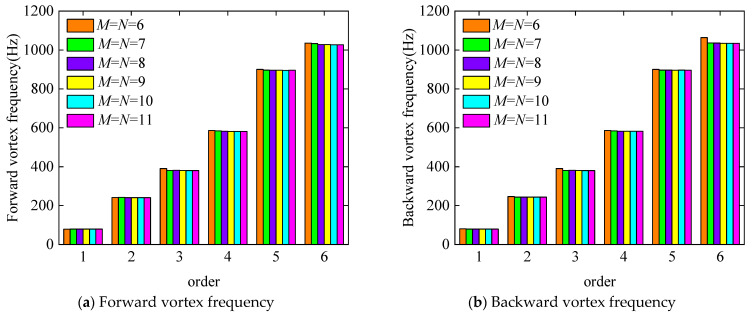
Convergence analysis of forward and backward vortex frequencies of the BFRC rotating annular plate with circle holes as the differential node increases.

In this paper, the convergence investigation for the BFRC rotating annular plate with holes was divided into two steps. First of all, as depicted in Figure 4, the convergence analysis concerning differential nodes *M* and *N* was executed. The isotropic material parameters were specified to be *E* = 210 GPa, *μ* = 0.3, and *ρ* = 7800 kg/m^3^. The geometrical properties were taken as *R_i_* = 0.04 m, *R_o_* = 0.2 m, *R_c_* = 0.12 m, *R_h_* = 0.03 m, *N_p_* = 4, *p* = 2, and *q* = 9, *h* = 0.008 m. The boundary condition chosen was a free boundary. The rotating speed was *Ω* = 500 rad/s.

As the differential nodes *M* and *N* increase, the first six-order forward and backward vortex frequencies of rotating annular plates with circle holes gradually decrease until they remain constant. The variation tendency to remain unchanged can be interpreted as the established theoretical model converges. Consequently, it can be concluded that the calculation results from the established theoretical model converge when the number of different nodes, *M* and *N*, reaches 10.

**Figure 5:**
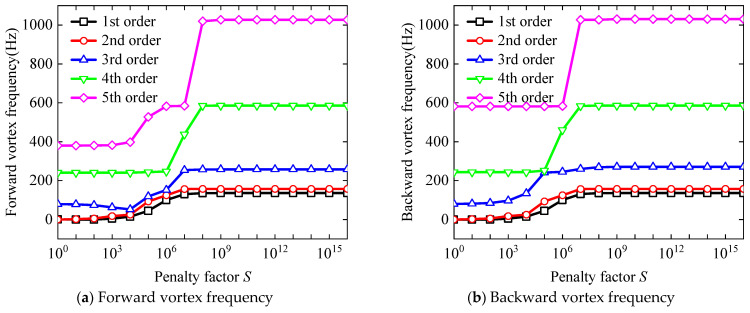
Convergence analysis of forward and backward vortex frequencies of the BFRC rotating annular plate with circle holes as the increase of penalty factor.

Then, the convergence analysis regarding the penalty factor *S* was presented, with the results depicted in Figure 5. These findings are consistent with the parameters outlined in Figure 4. Notably, the penalty factors with respect to the inner boundary of the rotating annular plate *S_φri_* = *S_φθi_* = 0 and the values of *S_ui_*, *S_vi_*, and *S_wi_* varied synchronously. The penalty factors with respect to the outer boundary of the BFRC rotating annular plate *S_uo_* = *S_vo_* = *S_wo_* = *S_φro_* = *S_φθo_* = 0.

As one can see from Figure 5, the first fifth-order forward and backward vortex frequencies of rotating annular plates with circle holes remain unchanged first, then increase evidently and finally remain unchanged with the increase of the penalty factors of *S_ui_*, *S_vi_*, and *S_wi_*. Based on the above variation tendencies of forward and backward vortex frequencies, it can be concluded that the calculation results of the theoretical model’s computational results converge as the value of penalty factor S increases to 10^13^. Thus, the penalty factor is chosen to be 10^13^ in the following discussion. In parallel, the penalty parameters corresponding to the boundary constraints for different perforated rotating annular plates are specified as follows. This article employs the letter F to denote a free boundary, S to signify a simply supported boundary, and the combinations SS and SD to distinguish between two types of simply supported boundary conditions. Additionally, the letter C is used to represent a clamped boundary condition.

(29) $$\begin{array}{l} F:S_{ui,o}=S_{vi,o}=S_{wi,o}=S_{\varphi ri,o}=S_{\varphi \theta i,o}=0\\ S:S_{ui,o}=S_{vi,o}=S_{wi,o}=10^{13},S_{\varphi ri,o}=S_{\varphi \theta i,o}=0\\ C:S_{ui,o}=S_{vi,o}=S_{wi,o}=S_{\varphi ri,o}=S_{\varphi \theta i,o}=10^{13}\\ SS:S_{ui,o}=S_{vi,o}=S_{wi,o}=S_{\varphi \theta i,o}=10^{13},S_{\varphi ri,o}=0\\ SD:S_{vi,o}=S_{wi,o}=10^{13},S_{ui,o}=S_{\varphi ri,o}=S_{\varphi \theta i,o}=0 \end{array}$$

### 3.2. Model Verification

After the above convergence analysis, the appropriate number of differential nodes and the optimal penalty factor values were determined. The model verification was executed to verify the accuracy, numerical stability, and reliability of the established BFRC rotating annular plate with holes. Model verification can be divided into three aspects: the comparison of works of literature, finite element simulation software comparison, and experimental comparison. The comparison of pieces of literature was performed first. Due to the vibration characteristic analysis of the established BFRC, rotating annular plates with holes are relatively rare in the published literature. Thus, circular plates with single eccentric holes were selected as objects, and the boundary conditions were chosen to be F-F. The isotropic material parameters are assumed as *E* = 210 GPa, *μ* = 0.3, and *ρ* = 7800 kg/m^3^. The geometrical parameters were assumed to be *R_i_* = 0 m, *R_o_* = 1 m, *R_c_* = 0.45 m, *R_h_* = 0.25 m, and *h* = 0.002 m. The formulations of dimensionless natural frequency *λ* are shown as follows. The comparison results can be seen in Table 1.

(30) $$\lambda ={\left(\omega^{2}R_{o}^{4}\rho h/D\right)}^{1/4},\omega =2\pi f,D=Eh^{3}/\left[12\left(1-\mu^{2}\right)\right]$$

Meanwhile, the expression of error is defined as follows.

(31) $$\mathrm{Error}=\left|\frac{\mathrm{Present}-\mathrm{Literature}}{\mathrm{Literature}}\right|\times 100\%$$

The comparative results displayed in Table 1 indicate a high level of agreement between the first six dimensionless frequencies derived from the current numerical model and those reported in the literature, with the maximum deviation being less than 1%.

Then, the comparison of finite element simulation software (ABAQUS) is given in the following discussion. The BFRC rotating annular plate with holes was selected as the study object, and a free-free (F-F) boundary condition was applied. The geometrical parameters were set as follows: *R_i_* = 0.02 m, *R_o_* = 0.1 m, *h* = 0.005 m, and *N_p_* = 4; for the circle hole, *R_c_* = 0.06 m, and *R_h_* = 0.025 m; and for the sector hole, *R_si_* = 0.04 m, *R_so_* = 0.08 m, and *R_d_* = 0.015 m.

As depicted in Table 2, a comparison of the vibrational frequencies and modes reveals a satisfactory alignment between the findings of the present model and those obtained from the ABAQUS finite element simulation software. The maximum error appeared in the first order, and the value was less than 1%. Meanwhile, the expression of the above error is defined as follows.

(32) $$\mathrm{Error}=\left|\frac{\mathrm{Present}-\mathrm{ABAQUS}}{\mathrm{ABAQUS}}\right|\times 100\%$$

**Figure 6:**
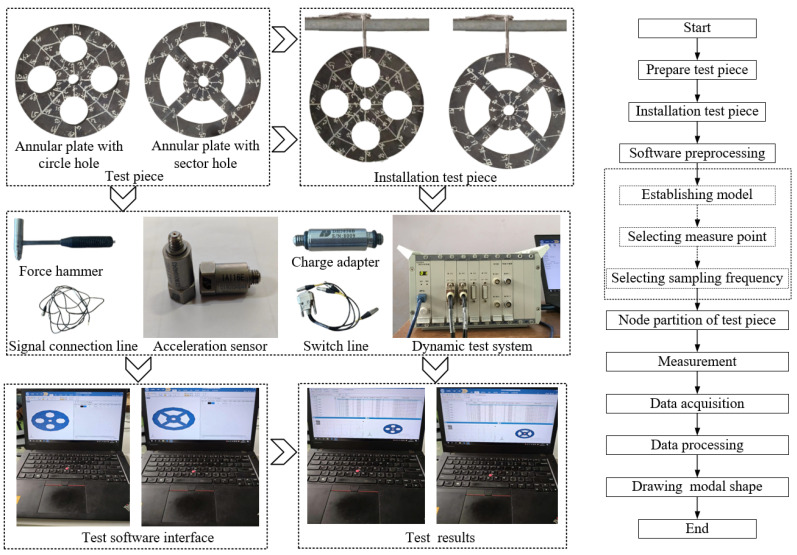
The experimental test flow chart of vibration and model shapes of isotropic annular plates with holes.

Finally, the comparison of experiments is displayed in the following discussion. The isotropic annular plate with holes was selected as the subject, and an F-F boundary condition was specified. The geometric parameters were set as follows: *R_i_* = 0.02 m, *R_o_* = 0.2 m, *h* = 0.008 m, and *N_p_* = 4; for circle holes, *R_c_* = 0.11 m, *R_h_* = 0.05 m; and for the sector holes, *R_si_* = 0.07 m, *R_so_* = 0.15 m, and *R_d_* = 0.02 m. The isotropic material parameters remain consistent with those presented in Table 1. The comparison results are presented in Table 3. Concurrently, the expression for error is defined as follows. The experiment of vibration and modal test are illustrated in Figure 6.

(33) $$\mathrm{Error}=\left|\frac{\mathrm{Present}-\mathrm{Experiment}}{\mathrm{Experiment}}\right|\times 100\%$$

The results presented in Table 3 indicate that the vibration frequencies and modes obtained in the present study exhibit preferably consistency with results from the experimental tests, with the maximum discrepancy not exceeding 2.7%. Based on these comparative findings, the accuracy, stability, and reliability of the established BFRC rotating annular plate with holes were corroborated from various angles. Consequently, the developed theoretical model is deemed suitable for analyzing the vibrational properties of the BFRC rotating annular plate with holes under diverse boundary conditions.

### 3.3. Parametric Study

The analysis of the vibrational characteristics of the BFRC rotating annular plate with holes in this section was conducted by examining the impact of material parameters, structural parameters, and boundary restraints on its vibration frequencies. The effect of the volume fraction on the vibration frequency of BFRC rotating annular plates was also investigated, with the results depicted in Figure 6. For BFRC rotating annular plate with circular holes, the geometrical parameters were chosen to be *R_i_* = 0.04 m, *R_o_* = 0.2 m, *R_c_* = 0.12 m, *R_h_* = 0.03 m, *N_p_* = 4, *p* = 2, *q* = 9, and *h* = 0.008 m. For the BFRC rotating annular plate with sector holes, the geometrical parameters were chosen to be *R_i_* = 0.02 m, *R_o_* = 0.1 m, *R_si_* = 0.04 m, *R_so_* = 0.08 m, *R_d_* = 0.015 m, *N_p_* = 4, *p* = 2, *q* = 9, and *h* = 0.008 m. The rotating speed was selected to be *Ω* = 1000 rad/s. Throughout the discussion, unless specified otherwise, the boundary constraints for BFRC rotating annular plates with holes are assumed to be S and F, respectively.

**Figure 7:**
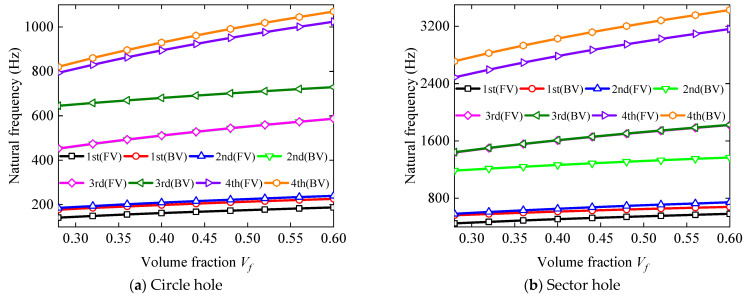
The influence of volume fraction on the natural frequency of the BFRC rotating annular plate with holes.

As described in Figure 7, the first four-order forward and backward vortex frequencies increase as the volume fraction increases. Meanwhile, the variation amplitudes of forward and backward vortex frequencies are more evident with the increase of frequency order. The findings of the analysis suggest that an increased volume fraction of fiber has the effect of raising the vibration frequencies of BFRC rotating annular plates with perforations.

**Figure 8:**
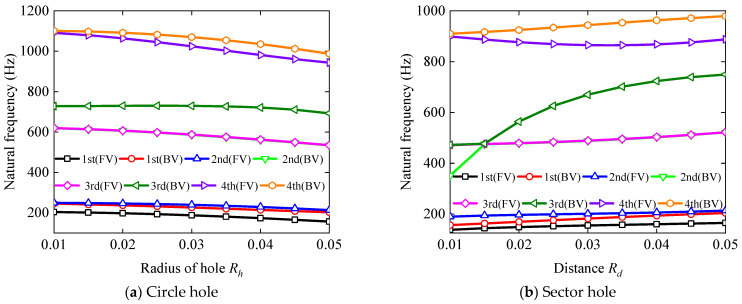
The influence of *R_h_* and *R_d_* on the natural frequency of the BFRC rotating annular plate with holes.

Next, the influence of the hole radius *R_h_* and the distance *R_d_* on the first four orders of vibration frequencies of BFRC rotating annular plates with holes were examined, with the findings obtained shown in Figure 8. The volume fraction of BFRC materials was selected as *V_f_* = 0.6. For BFRC rotating annular plate with circular holes, the geometrical parameters can be found in Figure 6 except for the radius of hole *R_h_*. For the BFRC rotating annular plate with sector holes, the geometrical parameters were selected to be *R_i_* = 0.04 m, *R_o_* = 0.2 m, *R_si_* = 0.08 m, *R_so_* = 0.16 m, *N_p_* = 4, *p* = 2, *q* = 9, and *h* = 0.008 m.

Figure 7 demonstrates that the first four forward and backward vortex frequencies of the BFRC rotating annular plate with circular holes decrease with an increasing radius of the hole *R_h_*. Conversely, for the plate with sector holes, the first four forward and backward vortex frequencies, except for the fourth order backward frequency, increase as the distance *R_d_* increases. It is observed that the rise in *R_h_* leads to a reduction in the vibration frequencies, while a rise in *R_d_* results in enhanced natural frequencies for the BFRC rotating annular plate with holes.

**Figure 9:**
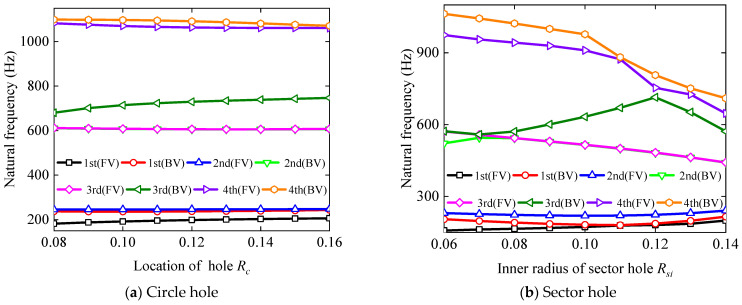
The influence of *R_c_* and *R_si_* on the natural frequency of the BFRC rotating annular plate with holes.

As depicted in Figure 9, the influences of the location of hole *R_c_* and inner radius of sector hole *R_si_* on the frequency of vibration for BFRC rotating annular plates with holes are examined. For BFRC rotating annular plate with circular holes, the geometrical parameters are consistent with Figure 8 except *R_c_*. For the BFRC rotating annular plate with sector holes, the geometrical parameters are the same as in Figure 7 except *R_si_* and *R_so_*; the increments of *R_si_* and *R_so_* are the same.

Figure 9 reveals that as the inner radius of the sector hole *R_si_* and the location of the hole *R_h_* increase, the first-order forward and backward vortex frequencies of BFRC rotating annular plates with holes exhibit an increase, whereas the high-order forward and backward vortex frequencies show a decrease. This underscores the significant impact of the choice of hole location on the vibration frequency of BFRC rotating annular plates with holes.

**Figure 10:**
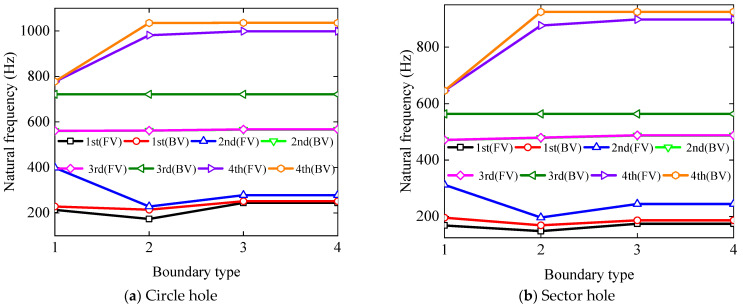
The influence of boundary conditions on the natural frequency of the BFRC rotating annular plate with holes.

Finally, the impact of different boundaries on the vibrational frequency of BFRC rotating annular plates with holes is discussed, with the results delineated in Figure 10. For the BFRC rotating annular plate with circular holes, the geometrical parameters are consistent with Figure 8, except *R_h_* = 0.04 m. For the BFRC rotating annular plate with sector holes, the geometrical parameters are the same as in Figure 8 except *R_si_* = 0.08 m and *R_so_* = 0.16 m. The boundary types 1, 2, 3, and 4 correspond to the boundary conditions SD, S, SS, and C, respectively. These boundary conditions exclusively apply to the inner boundary of the BFRC rotating annular plate with holes.

As one can see from Figure 10, the boundary condition *S* has an important influence on the first two-order forward vortex frequencies and the fourth-order vortex frequency of BFRC rotating annular plates with holes, in contrast to the other boundary conditions. The above variation tendency can be attributed to the influence of the degree of freedom *u*.

## 4. Conclusions

This study introduces a generalized numerical model for examining the vibrational properties of the BFRC rotating annular plate with perforations under diverse boundary constraints. The model employs the differential quadrature finite element method (DQFEM) in tandem with a coordinate mapping technique, all within the first-order shear deformation theory (FSDT). This study considered both circular and sector holes, utilizing a two-dimensional bidirectional orthogonal braided fiber-reinforced composite material to construct the BFRC rotating annular plate. The effectiveness of the current model was verified through comparative analysis with literature findings, ABAQUS simulations, and experimental data, ensuring its convergence, accuracy, numerical stability, and reliability. Numerical examples were utilized to conduct the vibrational characteristic analysis of perforated BFRC rotating annular plates. The representative conclusions are presented as follows.

(1) Incrementing the volume fraction for the global fiber within the BFRC material is advantageous for augmenting the vibration frequencies of BFRC rotating annular plates equipped with holes while simultaneously mitigating the detrimental effects introduced by the perforations.
(2) A rise in the area of holes leads to a diminution in the vibration frequencies of BFRC rotating annular plates with circular holes. Conversely, a relocation of the holes towards the outer radius of the plate results in an increase in the first-order natural frequencies, accompanied by a decrease in the higher-order natural frequencies.
(3) The presence or absence of constraints on the degree of freedom *u* significantly influences the vibrational properties of the BFRC rotating annular plate with holes, especially across different boundary conditions.

## Figures and Tables

**Table 1:** The comparison results of dimensionless natural frequencies of a circular plate with a single eccentric hole.

Method	Order					
	1	2	3	4	5	6
Present	3.206	4.531	4.752	5.783	6.090	6.242
Literature [11]	3.228	4.549	4.755	5.838	6.086	6.263
Literature [17]	3.187	4.521	4.740	5.758	6.060	6.256
Literature [22]	3.202	4.529	4.744	5.775	6.087	6.239
Error [11] (%)	0.682	0.396	0.063	0.942	0.066	0.335
Error [17] (%)	0.596	0.221	0.253	0.434	0.495	0.224
Error [22] (%)	0.131	0.049	0.160	0.147	0.044	0.045

**Table 2:** The comparison results of natural frequencies and mode shapes of the BFRC annular plate with holes.

Type	Method	Result	Order			
			1	2	3	4
Circle hole	Present	Mode shape	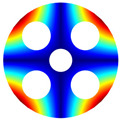	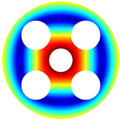	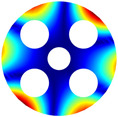	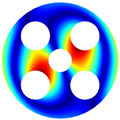
		Frequency	392.60	682.10	1021.94	1424.11
	ABAQUS	Mode shape	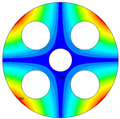	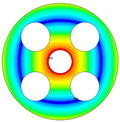	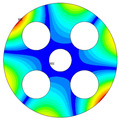	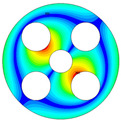
		Frequency	379.03	694.91	1023.98	1474.43
	Error (%)		3.58	1.84	0.20	3.41
Sector hole	Present	Mode shape	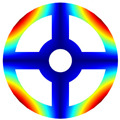	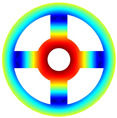	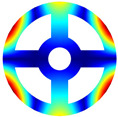	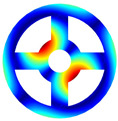
		Frequency	365.44	621.88	933.93	1314.19
	ABAQUS	Mode shape	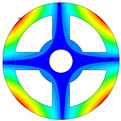	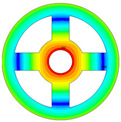	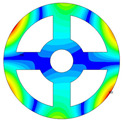	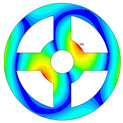
		Frequency	354.09	622.13	918.34	1314.89
	Error(%)		3.21	−0.04	1.70	−0.05

**Table 3:** The comparison results of natural frequencies and mode shapes of the isotropic annular plate with holes.

Type	Method	Result	Order			
			1	2	3	4
Circle hole	Present	Mode shape	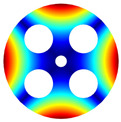	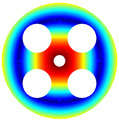	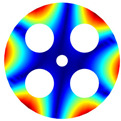	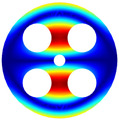
		Frequency	218.64	331.99	534.76	847.42
	Experiment	Mode shape	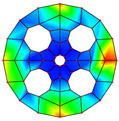	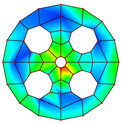	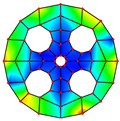	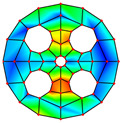
		Frequency	217.01	327.98	525.56	830.14
	Error(%)		0.75	1.22	1.75	2.08
Sector hole	Present	Mode shape	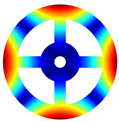	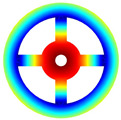	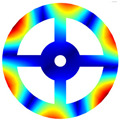	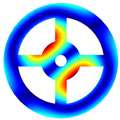
		Frequency	207.19	291.29	498.92	753.27
	Experiment	Mode shape	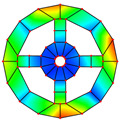	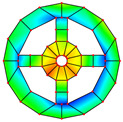	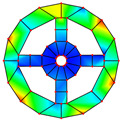	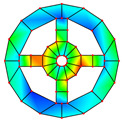
		Frequency	203.16	287.01	485.94	734.95
	Error(%)		1.98	1.49	2.67	2.49

## Data Availability

The original contributions presented in the study are included in the article, further inquiries can be directed to the corresponding authors.

## Appendix A. The Annular Plate Element Mass Matrix $M_{e}^{s}$

$$M_{e}^{s}=\left[\begin{array}{cc} M_{e11}^{s} & M_{e12}^{s}\\ M_{e21}^{s} & M_{e22}^{s} \end{array}\right]\;M_{e11}^{s}=\left[\begin{array}{cccccc} {\hat{I}}_{1}r{\hat{C}}_{2} & 0 & 0 & 0 & 0 & 0\\ 0 & {\hat{I}}_{1}r{\hat{C}}_{2} & 0 & 0 & 0 & 0\\ 0 & 0 & {\hat{I}}_{1}r{\hat{C}}_{2} & 0 & 0 & 0\\ 0 & 0 & 0 & {\hat{I}}_{1}r^{3}{\hat{C}}_{2} & 0 & 0\\ 0 & 0 & 0 & 0 & {\hat{I}}_{3}r{\hat{C}}_{2}+{\hat{I}}_{1}r^{3}\sin\theta {\hat{C}}_{2} & 0\\ 0 & 0 & 0 & 0 & 0 & {\hat{I}}_{3}r{\hat{C}}_{2}+{\hat{I}}_{1}r^{3}\sin\theta {\hat{C}}_{2} \end{array}\right]\;M_{e12}^{s}=M_{e21}^{sT}=\left[\begin{array}{ccccc} {\hat{I}}_{1}rC_{2} & 0 & 0 & 0 & 0\\ 0 & {\hat{I}}_{1}r\cos\theta C_{2} & -{\hat{I}}_{1}r\sin\theta C_{2} & 0 & 0\\ 0 & {\hat{I}}_{1}r\sin\theta C_{2} & {\hat{I}}_{1}r\cos\theta C_{2} & 0 & 0\\ 0 & 0 & {\hat{I}}_{1}r^{2}C_{2} & 0 & 0\\ {\hat{I}}_{1}r^{2}\sin\theta C_{2} & 0 & 0 & {\hat{I}}_{3}r\cos\theta C_{2} & -{\hat{I}}_{3}r\sin\theta C_{2}\\ -{\hat{I}}_{1}r^{2}\cos\theta C_{2} & 0 & 0 & {\hat{I}}_{3}r\sin\theta C_{2} & {\hat{I}}_{3}r\cos\theta C_{2} \end{array}\right]\;M_{e22}^{s}=\left[\begin{array}{ccccc} I_{1}rC_{2} & 0 & 0 & 0 & 0\\ 0 & I_{1}rC_{2} & 0 & 0 & 0\\ 0 & 0 & I_{1}rC_{2} & 0 & 0\\ 0 & 0 & 0 & I_{3}rC_{2} & 0\\ 0 & 0 & 0 & 0 & I_{3}rC_{2} \end{array}\right]$$

where the symbol $I_{i}$ denotes the diagonal matrix, which consists of inertia element *I_i_*; The symbols *r* and ***θ*** indicate the diagonal matrices, which are composed of differential node coordinate *r_ij_* and *θ_ij_*, respectively; The symbols ${\hat{I}}_{i}$ and ${\hat{C}}_{2}$ express the row and column vectors, which can be determined by ***I**_i_* and ***C***_2_. The specific expression of inertia element *I_i_* is given as follows.

$$\left(I_{1},I_{2},I_{3}\right)=\rho \int_{-\frac{h}{2}}^{\frac{h}{2}}\left(1,x,x^{2}\right)dx,\left(i,j=1,2,3\right)$$

## Appendix B. The Annular Plate Element Gyroscopic Matrix $G_{e}^{s}$

$$G_{e}^{s}=\left[\begin{array}{cc} G_{e11}^{s} & G_{e12}^{s}\\ G_{e21}^{s} & G_{e22}^{s} \end{array}\right]\;G_{e11}^{s}=2\Omega \left[\begin{array}{cccccc} 0 & 0 & 0 & 0 & 0 & 0\\ 0 & 0 & -{\hat{I}}_{1}r{\hat{C}}_{2} & 0 & 0 & 0\\ 0 & {\hat{I}}_{1}r{\hat{C}}_{2} & 0 & 0 & 0 & 0\\ 0 & 0 & 0 & 0 & 0 & 0\\ 0 & 0 & 0 & 0 & 0 & -{\hat{I}}_{3}r{\hat{C}}_{2}-{\hat{I}}_{1}r^{3}\sin^{2}\theta {\hat{C}}_{2}\\ 0 & 0 & 0 & 0 & {\hat{I}}_{3}r{\hat{C}}_{2}-{\hat{I}}_{1}r^{3}\sin^{2}\theta {\hat{C}}_{2} & 0 \end{array}\right]\;G_{e12}^{s}=-2\Omega \left[\begin{array}{ccccc} 0 & 0 & 0 & 0 & 0\\ 0 & {\hat{I}}_{1}r\sin\theta C_{2} & {\hat{I}}_{1}r\cos\theta C_{2} & 0 & 0\\ 0 & -{\hat{I}}_{1}r\cos\theta C_{2} & {\hat{I}}_{1}r\sin\theta C_{2} & 0 & 0\\ 0 & -2{\hat{I}}_{1}r^{2}C_{2} & 0 & 0 & 0\\ {\hat{I}}_{1}r^{2}\cos\theta C_{2} & 0 & 0 & {\hat{I}}_{3}r\sin\theta C_{2} & {\hat{I}}_{3}r\cos\theta C_{2}\\ {\hat{I}}_{1}r^{2}\sin\theta C_{2} & 0 & 0 & -{\hat{I}}_{3}r\cos\theta C_{2} & {\hat{I}}_{3}r\sin\theta C_{2} \end{array}\right]\;G_{e21}^{s}=2\Omega \left[\begin{array}{cccccc} 0 & 0 & 0 & 0 & -I_{1}r^{2}\cos\theta {\hat{C}}_{2} & -I_{1}r^{2}\sin\theta {\hat{C}}_{2}\\ 0 & I_{1}r\sin\theta {\hat{C}}_{2} & -I_{1}r\cos\theta {\hat{C}}_{2} & 0 & 0 & 0\\ 0 & I_{1}r\cos\theta {\hat{C}}_{2} & I_{1}r\sin\theta {\hat{C}}_{2} & 0 & 0 & 0\\ 0 & 0 & 0 & 0 & I_{3}r\sin\theta {\hat{C}}_{2} & -I_{3}r\cos\theta {\hat{C}}_{2}\\ 0 & 0 & 0 & 0 & I_{3}r\cos\theta {\hat{C}}_{2} & I_{3}r\sin\theta {\hat{C}}_{2} \end{array}\right]\;G_{e22}^{s}=2\Omega \left[\begin{array}{ccccc} 0 & 0 & 0 & 0 & 0\\ 0 & 0 & -I_{1}rC_{2} & 0 & 0\\ 0 & I_{1}rC_{2} & 0 & 0 & 0\\ 0 & 0 & 0 & 0 & -I_{3}rC_{2}\\ 0 & 0 & 0 & I_{3}rC_{2} & 0 \end{array}\right]$$

## Appendix C. The Annular Plate Element Damp Matrix $C_{e}^{s}$

$$C_{e}^{s}=\left[\begin{array}{cc} C_{e11}^{s} & C_{e12}^{s}\\ C_{e21}^{s} & C_{e22}^{s} \end{array}\right]\;C_{e11}^{s}=\Omega^{2}\left[\begin{array}{cccccc} 0 & 0 & 0 & 0 & 0 & 0\\ 0 & {\hat{I}}_{1}r{\hat{C}}_{2} & 0 & 0 & 0 & 0\\ 0 & 0 & {\hat{I}}_{1}r{\hat{C}}_{2} & 0 & 0 & 0\\ 0 & 0 & 0 & 0 & 0 & 0\\ 0 & 0 & 0 & 0 & {\hat{I}}_{3}r{\hat{C}}_{2}-{\hat{I}}_{1}r^{3}\sin^{2}\theta {\hat{C}}_{2} & 0\\ 0 & 0 & 0 & 0 & 0 & {\hat{I}}_{3}r{\hat{C}}_{2}-{\hat{I}}_{1}r^{3}\cos^{2}\theta {\hat{C}}_{2} \end{array}\right]\;C_{e12}^{s}=C_{e21}^{sT}=\Omega^{2}\left[\begin{array}{ccccc} 0 & 0 & 0 & 0 & 0\\ 0 & {\hat{I}}_{1}r\cos\theta C_{2} & -{\hat{I}}_{1}r\sin\theta C_{2} & 0 & 0\\ 0 & {\hat{I}}_{1}r\sin\theta C_{2} & {\hat{I}}_{1}r\cos\theta C_{2} & 0 & 0\\ 0 & 0 & 0 & 0 & 0\\ -{\hat{I}}_{1}r^{2}\sin\theta C_{2} & 0 & 0 & {\hat{I}}_{3}r\cos\theta C_{2} & -{\hat{I}}_{3}r\sin\theta C_{2}\\ {\hat{I}}_{1}r^{2}\cos\theta C_{2} & 0 & 0 & {\hat{I}}_{3}r\sin\theta C_{2} & {\hat{I}}_{3}r\cos\theta C_{2} \end{array}\right]\;G_{e22}^{s}=\Omega^{2}\left[\begin{array}{ccccc} 0 & 0 & 0 & 0 & 0\\ 0 & I_{1}rC_{2} & 0 & 0 & 0\\ 0 & 0 & I_{1}rC_{2} & 0 & 0\\ 0 & 0 & 0 & I_{3}rC_{2} & 0\\ 0 & 0 & 0 & 0 & I_{3}rC_{2} \end{array}\right]$$

## Appendix D. The Annular Plate Element Stiffness Matrix $K_{e}^{s}$

$$K_{e}^{s}=\left[\begin{array}{ccccc} k_{11} & k_{12} & k_{13} & k_{14} & k_{15}\\ k_{12}^{T} & k_{22} & k_{23} & k_{24} & k_{25}\\ k_{13}^{T} & k_{32}^{T} & k_{33} & k_{34} & k_{35}\\ k_{14}^{T} & k_{24}^{T} & k_{34}^{T} & k_{44} & k_{45}\\ k_{15}^{T} & k_{25}^{T} & k_{35}^{T} & k_{45}^{T} & k_{55} \end{array}\right]\;\begin{array}{l} k_{11}=\kappa A_{44}A_{2}^{T}rC_{2}A_{2}+\kappa A_{55}B_{2}^{T}\frac{1}{r}C_{2}B_{2}\\ k_{12}=k_{13}=0\\ k_{14}=\kappa A_{44}B_{2}^{T}C_{2}\\ k_{15}=\kappa A_{55}A_{2}^{T}rC_{2} \end{array}\;\begin{array}{l} k_{22}=A_{11}A_{2}^{T}rC_{2}A_{2}+A_{22}\frac{1}{r}C_{2}+A_{12}\left(C_{2}A_{2}+A_{2}^{T}C_{2}\right)+A_{66}B_{2}^{T}\frac{1}{r}C_{2}B_{2}\\ k_{23}=A_{22}\frac{1}{r}C_{2}B_{2}+A_{12}A_{2}^{T}C_{2}B_{2}+A_{66}B_{2}^{T}\left(C_{2}A_{2}-\frac{1}{r}C_{2}\right)\\ k_{24}=-B_{22}\frac{1}{r}C_{2}B_{2}-B_{12}A_{2}^{T}C_{2}B_{2}+B_{66}B_{2}^{T}\left(\frac{1}{r}C_{2}-C_{2}A_{2}\right)\\ k_{25}=B_{11}A_{2}^{T}rC_{2}A_{2}+B_{22}\frac{1}{r}C_{2}+B_{12}\left(C_{2}A_{2}+A_{2}^{T}C_{2}\right)+B_{66}B_{2}^{T}\frac{1}{r}C_{2}B_{2} \end{array}\;\begin{array}{l} k_{33}=A_{22}B_{2}^{T}\frac{1}{r}C_{2}B_{2}+A_{66}\left(A_{2}^{T}rC_{2}A_{2}-C_{2}A_{2}-A_{2}^{T}C_{2}+\frac{1}{r}C_{2}\right)\\ k_{34}=-B_{22}B_{2}^{T}\frac{1}{r}C_{2}B_{2}+B_{66}\left(C_{2}A_{2}+A_{2}^{T}C_{2}-A_{2}^{T}rC_{2}A_{2}-\frac{1}{r}C_{2}\right)\\ k_{35}=B_{22}B_{2}^{T}\frac{1}{r}C_{2}+B_{12}B_{2}^{T}C_{2}A_{2}+B_{66}\left(A_{2}^{T}-\frac{1}{r}\right)C_{2}B_{2} \end{array}\;\begin{array}{l} k_{44}=D_{22}B_{2}^{T}\frac{1}{r}C_{2}B_{2}+D_{66}\left(A_{2}^{T}r-E\right)C_{2}A_{2}+D_{66}\left(\frac{1}{r}-A_{2}^{T}\right)C_{2}+\kappa A_{44}rC_{2}\\ k_{45}=-D_{22}B_{2}^{T}\frac{1}{r}C_{2}-D_{12}B_{2}^{T}C_{2}A_{2}+D_{66}\left(\frac{1}{r}-A_{2}^{T}\right)C_{2}B_{2} \end{array}\;k_{55}=D_{11}A_{2}^{T}rC_{2}A_{2}+D_{22}\frac{1}{r}C_{2}+D_{12}\left(C_{2}A_{2}+A_{2}^{T}C_{2}\right)+D_{66}B_{2}^{T}\frac{1}{r}C_{2}B_{2}+\kappa A_{55}rC_{2}$$

The symbols *A_ij_*, *B_ij_*, and *D_ij_* denote the stiffness coefficients, and the corresponding expression can be written as follows:

$$\left(A_{ij},B_{ij},D_{ij}\right)=\int_{-\frac{h}{2}}^{\frac{h}{2}}Q_{ij}\left(1,x,x^{2}\right)dx,\left(i,j=1,2,4,5,6\right)$$
